# Classic but unexpected: a case of Jefferson fracture

**DOI:** 10.1007/s12024-020-00333-1

**Published:** 2020-11-05

**Authors:** Jean-Loup Gassend, Mohamed Yassine Braham, Raquel Vilarino, Virginie Magnin

**Affiliations:** Centre Universitaire Romand de Médecine Légale, Centre Hospitalier Universitaire Vaudois, Lausanne, Switzerland

**Keywords:** Jefferson fracture, Atlas fracture, Skull apex, Forensic

## Abstract

A man was found lying dead next to a ladder, with only a laceration surrounded by an abrasion visible upon external examination. No skull fractures were palpable. A CT scan and MRI showed a Jefferson fracture of the atlas, associated to a posterior displacement of the skull, a fracture of the dens of the axis, and fractures of the bodies of C5 and C6. Jefferson fractures typically result from a blow to the apex of the skull. In such cases, forensic pathologists should suspect the existence of a Jefferson fracture, particularly when no severe injuries are visible externally.

## Introduction

The superior articular surfaces of the atlas face upwards and inwards, and its inferior articular surfaces downwards and inwards. The articular surfaces of the occipital condyles and of the axis are oriented correspondingly, ensuring a close fit between all three bones. However, in situations in which a powerful axial load causes a compression of the atlas between the occipital condyles and the axis, this orientation of the atlas’ articular surface causes its two lateral masses to be pushed outwards. If the load is powerful enough, a fracture of the anterior and posterior arches of the atlas will ensue, in what is known as a Jefferson fracture [1]. This compressive mechanism typically occurs when diving into shallow water onto the vertex, or when motorists are forcefully thrown against the roof of their car during motor vehicle accidents [2]. Because of the powerful force needed to produce a Jefferson fracture, it is common for it to be associated with other cervical or head injuries [3].

## Case report

**Fig. 1:**
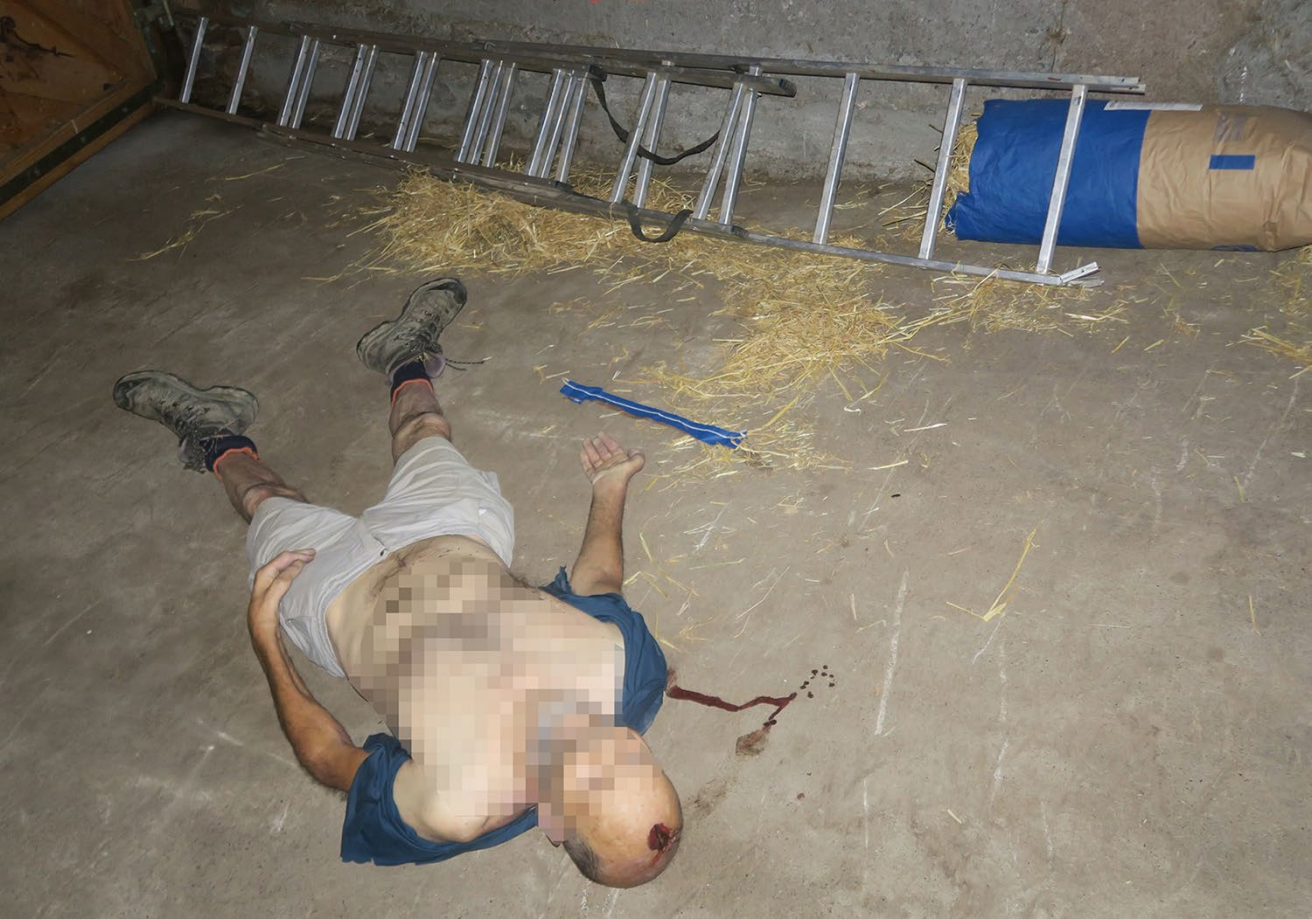
A cliché scene of death for a Jefferson fracture: note the laceration and abrasion near the apex and the broken ladder in the background

**Fig. 2:**
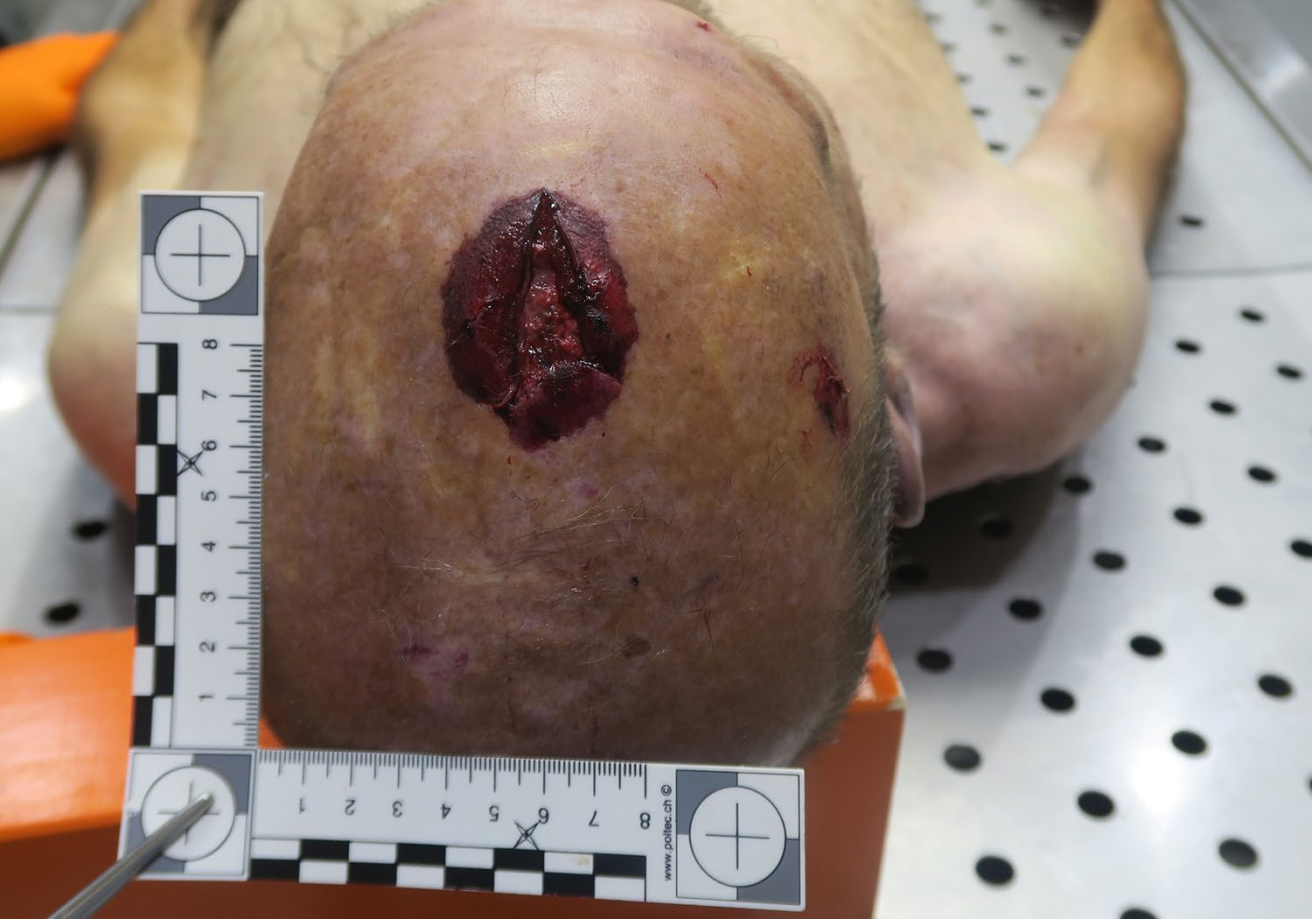
Close up view of the laceration and abrasion of the scalp once cleaned. Note that they are located forward of the apex

A septuagenarian with a history of loss of balance and dizziness told his wife that he was going to his barn to get some hay, which was stocked in the barn on a wooden structure approximately three meters high. When the man did not return, his family got worried and his son went to look for him. The son found him lying unconscious on the ground in the barn, near the wooden structure for stowing hay. A ladder with a broken foot was lying next to him, and a small quantity of blood was visible on the ground. The son called an ambulance and cardio-pulmonary resuscitation was attempted, however the man was declared dead at the scene (Fig. 1). A forensic pathologist was called to the site, mainly noting a laceration of the scalp measuring 4.5 × 2 cm on the midline in the fronto-parietal area, surrounded by an oval abrasion measuring 5 × 3.5 cm (Fig. 2). The forensic pathologist was puzzled that no skull fractures seemed to be palpable and determined that no obvious cause of death was visible. The body was transferred to the forensic institute for an external examination and imaging. The external examination did not bring to light any new findings, apart from a few minor bruises and abrasions that were noted elsewhere on the body.

### Post mortem CT and MRI findings

**Fig. 3:**
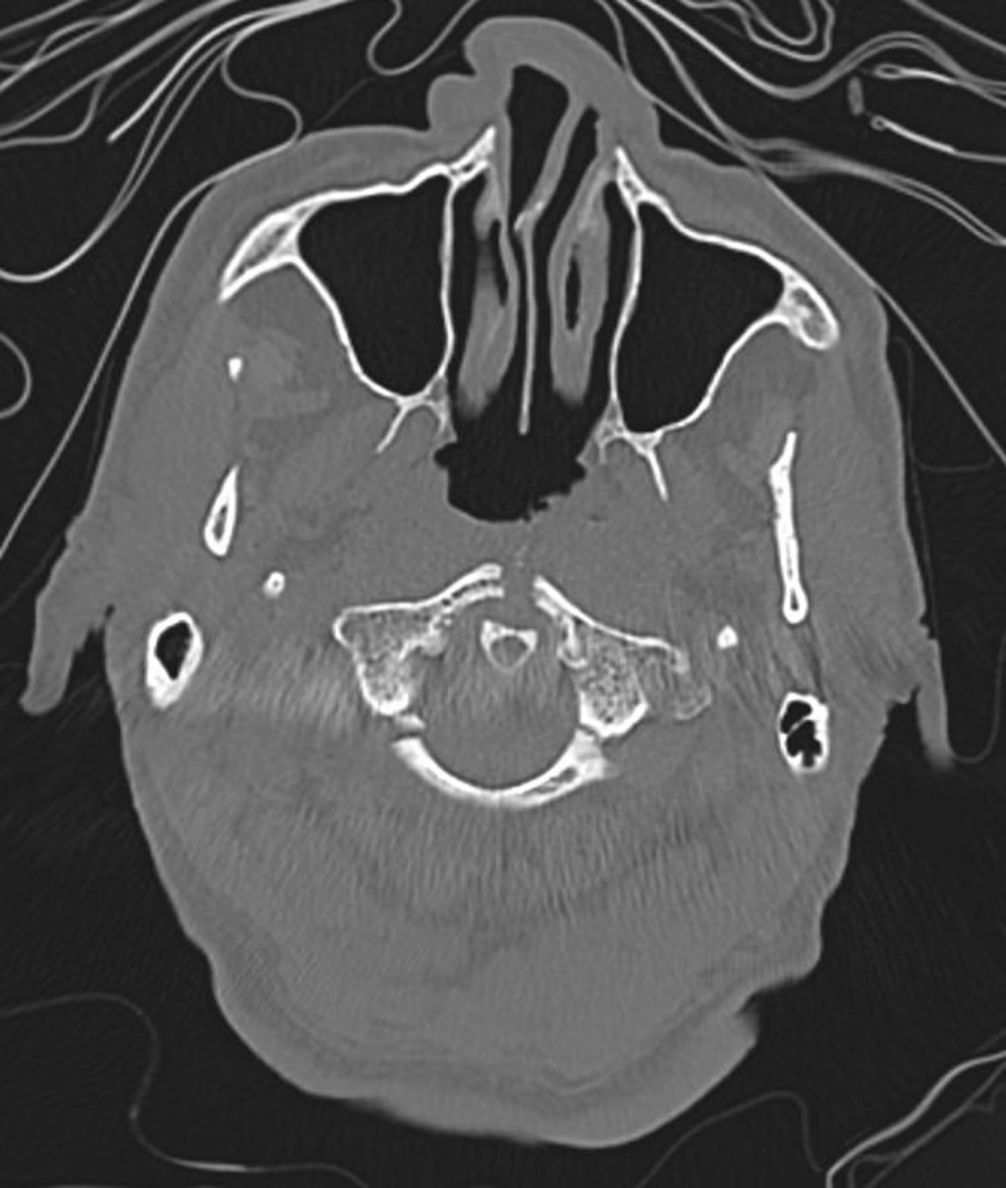
Fractures of the anterior and the posterior arches of the atlas

**Fig. 4:**
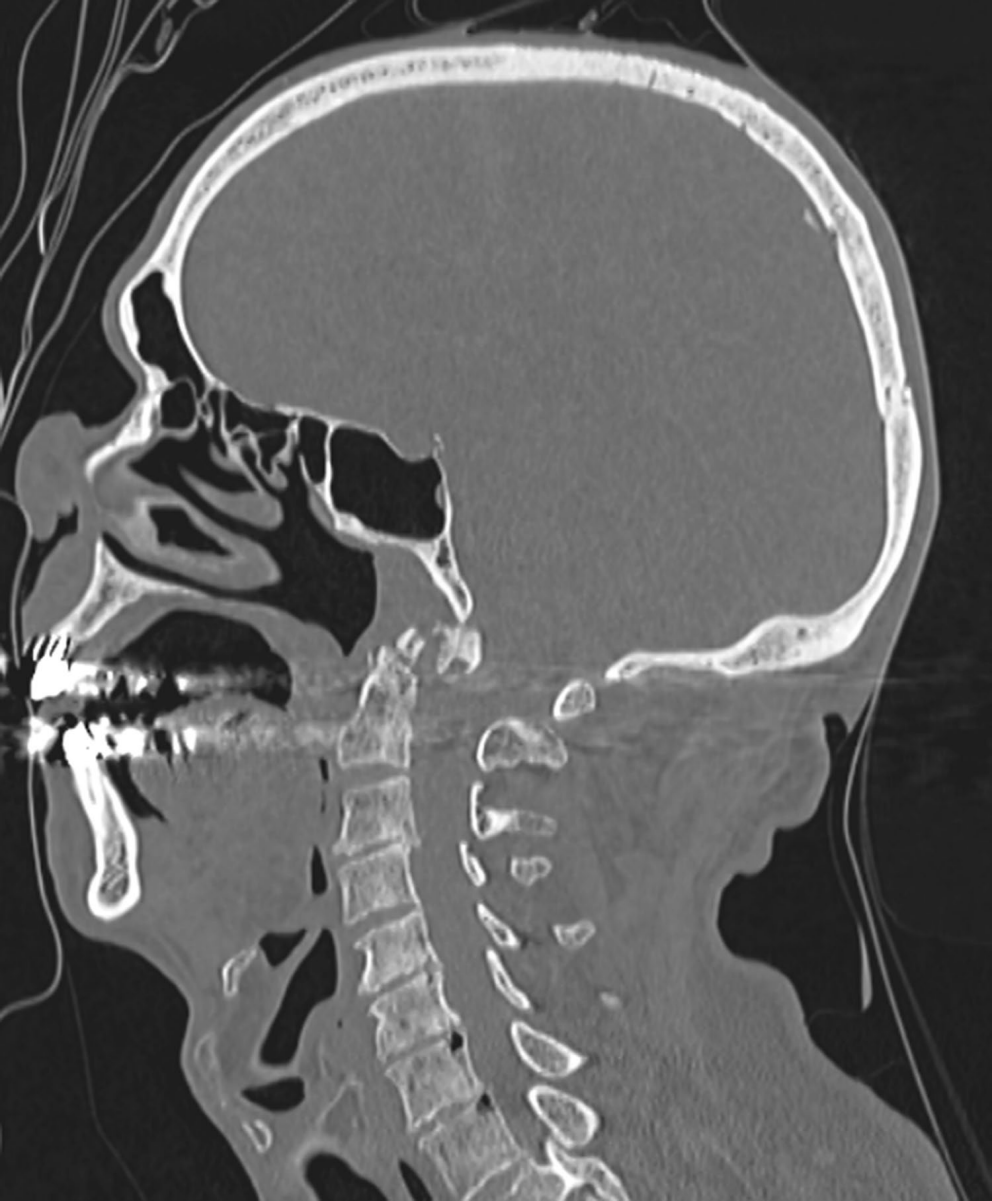
Fracture of the odontoid process of the axis with posterior displacement of the foramen magnum and skull

A CT scan as well as an MRI were performed, revealing (Figs. 3 and 4) an acute midline fracture of the anterior arch of the atlas, two acute fractures of the posterior arch, near the lateral arches, an acute fracture of the odontoid process of the axis with posterior displacement of the dens, a posterior displacement of the foramen magnum and skull as compared with the cervical vertebrae, and acute fractures of the bodies of C5 and C6.

In other words, the imaging showed a Jefferson’s fracture associated to a posterior displacement of the skull, a fracture of the dens and fractures of the bodies of C5 and C6.

## Discussion

The external examination and imaging results made it clear that the laceration on the man’s skull were most likely due to a fall on the head. The impact point being located slightly forward of the apex, the forces transmitted down the cervical spine would not have been purely vertical, but would also have had a posterior component. This posterior component probably explains the fracture of the dens of the axis and the posterior displacement of the skull, while the vertical component of the forces provide a classical explanation for the atlas fractures. Although no autopsy was performed, the radiological examinations enabled a probable cause of death to be documented. In cases when a fall on or near the apex of the head is suspected, the possibility of a Jefferson fracture should be kept in mind, particularly if no other signs of severe lesions are visible on the body.
